# Key actors’ perspectives on cost-effectiveness analysis in Uganda: a cross-sectional survey

**DOI:** 10.1186/s12913-014-0539-8

**Published:** 2014-11-04

**Authors:** Jackson S Musuuza, Mendel E Singer, Anna M Mandalakas, Achilles Katamba

**Affiliations:** Institute for Clinical and Translational Research, University of Wisconsin School of Medicine and Public Health, Madison, Wisconsin USA; Department of Epidemiology and Biostatistics, Case Western Reserve University, Cleveland, Ohio USA; Department of Pediatrics, Baylor College of Medicine, Houston, Texas USA; Department of Medicine, Makerere University College of Health Sciences, Kampala, Uganda

**Keywords:** Cost-effectiveness analysis, Healthcare in Uganda, Constrained resources

## Abstract

**Background:**

Cost effectiveness analysis (CEA) is a useful tool for allocation of constrained resources, yet CEA methodologies are rarely taught or implemented in developing nations. We aimed to assess exposure to, and interest in CEA, and identify barriers to implementation in Uganda.

**Methods:**

A cross-sectional survey was carried out in Uganda using a newly developed self-administered questionnaire (via online and paper based approaches), targeting the main health care actors as identified by a previous study.

**Results:**

Overall, there was a 68% response rate, with a 92% (69/75) response rate among the paper-based respondents compared to a 40% (26/65) rate with the online respondents. Seventy eight percent (74/95) of the respondents had no exposure to CEA. None of those with a master of medicine degree had any CEA exposure, and 80% of technical officers, who are directly involved in policy formulation, had no CEA exposure. Barriers to CEA identified by more than 50% of the participants were: lack of information technology (IT) infrastructure (hardware and software); lack of local experts in the field of CEA; lack of or limited local data; limited CEA training in schools; equity or ethical issues; and lack of training grants incorporating CEA. 93% reported a lot of interest in learning to conduct CEA, and over 95% felt CEA was important for clinical decision making and policy formulation.

**Conclusions:**

Among health care actors in Uganda, there is very limited exposure to, but substantial interest in conducting CEA and including it in clinical decision making and health care policy formation. Capacity to undertake CEA needs to be built through incorporation into medical training and use of regional approaches.

## Background

In the past 20 years, cost-effectiveness analysis (CEA) has become increasingly popular as a means of assessing the value of health care treatments. CEA endeavors to guide decision making such as to compare the health benefits of competing treatments within the context of cost. While these methods have been refined and gained traction in developed nations, there has been little use of CEA in developing nations [1]. Countries with scarce health care resources would logically stand to gain most from these methods to optimize allocation of those scarce resources. In Sub-Saharan Africa (SSA), adverse factors ranging from civil wars and extreme poverty to high rates of infectious disease strain health care budgets and delivery systems, making optimal allocation of health care especially important. Taken in the context of an expected doubling of the population in the next 25 years [2], it becomes even more urgent that CEA be more widely conducted and used to inform policy and practice. Accordingly, the World Health Organization (WHO) recommends incorporation of CEA in policy formulation and practice [3]. Despite all this, CEA publications in developing countries are few [1], and some have employed inconsistent and often flawed methodology [4].

Additional potential barriers to the conduct and impact of CEA in developing nations include lack of trained local personnel, lack of local data, and poor infrastructure [5]. Other hypothesized barriers include a shortage of CEA experts and lack of familiarity with CEA among policy makers and opinion leaders.

Despite the presence of barriers, evidence from developing countries continues to show that CEA is perceived as important for decision making. A study done among Ugandan health workers and policy makers showed that about 90% of all respondents recognized both disease severity and cost effectiveness as very important in health care decision making [6]. However, we have not come across studies which have attempted to document key actors’ perspectives in regard to the barriers which attenuate the influence of CEA in policy formulation and practice in Sub-Saharan Africa.

In a commentary on the role of pharmacoeconomics in developing countries that was recently published in *PharmacoEconomics*, Babar and Scahill [7] argue that “there is a need for a model that could aid in determining the perceived need and benefits of using pharmacoeconomics in formulary development in a given developing country”. By providing primary research on the exposure, interest and barriers to CEA in the context of a developing Sub-Saharan African nation, our study attempts to make a contribution to this effect.

This study aims to identify barriers to the conduct of CEA and its impact on policy and practice in a Sub-Saharan African country, Uganda. It also assesses the interest in and exposure to CEA methods, as well as the frequency of use of CEA in actual health care policy and practice.

## Methods

We carried out a cross sectional survey of the different health care delivery actors in Uganda. Self-administered questionnaires were used. In order to increase the response rate, we used both paper-based and online questionnaires. After two reminders (chosen arbitrarily) were given following no response on the paper version, we would send the online version. This approach was not effective and was applied to only six participants. We eventually opted to invite all those who had not completed the paper version to complete an online questionnaire. The online questionnaires were designed and administered using REDCap Survey Software (version 1.3.10; Vanderbilt University, Tennessee, USA).

Questionnaires were sent out to health care workers; technical officers at the Ministry of Health (these are directly involved in policy formulation), post-graduate students training in health- related fields, and professors in the medical schools.

Health care workers and students were drawn from the two main teaching hospitals namely: Mulago teaching hospital for Makerere University and Mbarara University teaching hospital for Mbarara University of Science and Technology. The two hospitals were selected because they are the two main teaching hospitals in Uganda; it is the students trained in these hospitals that end up being the main decision makers in health care.

The study participants were selected as follows: For the Ministry of Health Officials, we contacted a senior officer and after we described the study to him, he advised us to send questionnaires to specific individuals from 3 departments that are involved in economic evaluations, namely Planning − 5 respondents; Clinical Services-5 respondents; and Community Health- 5 respondents. For the students in the two medical schools, we obtained a list of names of postgraduate students from the program administrators and randomly selected those to whom the questionnaires were sent. We obtained a similar list for health care workers at the level of medical officer and above and randomly selected respondents. We also sent questionnaires to directors or deputy directors of Non-governmental organizations involved in health care delivery. All participants were fluent in English, so it was not necessary to provide translations.

Data was collected between July and October 2010. Bi-weekly e-mail reminders were sent to participants.

The study received IRB approval from the Case Western Reserve University Institutional Review Board (IRB) and the Makerere University Faculty of Medicine Scientific and Ethics committee, with final approval from the Ugandan National Council of Science and Technology. This study was conducted under standard ethical practices.

### Statistical analysis

Data were entered online using REDCap Survey, and analyzed using SAS (version 9.2; SAS Institute,Cary North Carolina,USA). We carried out descriptive analyses looking at the demographic characteristics of respondents such as sex, education and work place.

Exposure to CEA was treated as a binary variable i.e. some exposure (for those who had some or a lot of exposure during their training, current or past) and no exposure (for those who had no exposure). Some exposure as used in this paper refers to whether someone was exposed to CEA through their current or past training and/or through any other setting such as short term trainings, conferences/workshops, grand rounds, online material and journal clubs.

A lot of exposure refers to someone who had classroom training in CEA and he/she is currently actively carrying out or involved in CEA research. No exposure means someone who did not have any exposure to CEA prior to this study.

All questions involving a five-point scale of strongly agree, agree, neutral, disagree and strongly disagree, were re-coded as agree (strongly agree and agree) and disagree (neutral, disagree and strongly disagree). Neutral was included in the disagree category to bias results against the null hypothesis.

Variables concerning the level of interest in conducting or learning to conduct CEA were coded as: a lot of interest (a lot of interest) and not a lot of interest (little, some or no interest).

Questions involving the frequency of use of CEA were coded as: always, sometimes, and rarely or never.

In order to test associations, we did a chi-square goodness of fit test to test whether the observed proportions for ‘agree’ or ‘disagree’ were different when a given question concerned policy versus when the same question concerned clinical practice.

In addition, we did chi-square tests looking at the association between interest in conducting or learning to conduct CEA versus exposure to CEA. We also asked participants about potential barriers to CEA and their solutions.

## Results

A total of 134 questionnaires were sent out. Of these 75 (57.2%) were paper-based and 65 (46.4%) were online. Six of the 65 online ones were from those who received the paper-based version and never responded. The remaining 59 were all sent directly online. Of the 75 paper- based ones, 69 (92.3%) were returned, but only 26 (40%) of the 65 online participants responded. Results are derived from a total of 95 (68%) returned questionnaires.

Majority of the respondents were employees of tertiary/referral hospitals and universities. These accounted for 34% and 28% respectively. The mean age of respondents was 36 years and males represented 61% of all respondents (Table 1).

**Table 1:** **Demographic characteristics of the survey respondents**

**Characteristic**	**N (%)**
**Sex**	
Male	58 (61.1)
Female	36 (38.8)
Missing	1 (1.1)
**Work-** **place**	
Ministry of Health	15 (15.7)
Tertiary/referral hospital	32 (33.7)
University	27 (28.4)
NGO	20 (21.1)
Missing	1 (1.1)
**Training completed**	
Bachelor of medicine/MD	59 (62.1)
Postgraduate training/MMED	8 (8.4)
MS/MPH	12 (12.6)
PhD	2 (2.1)
*Other	13 (13.7)
Missing	1(1.1)
**Current training**	
Postgraduate training/MMED	17 (18.9)
MS/MPH	16 (16.8)
PhD in a health related field (e.g. epidemiology or Health Services Research)	23 (24.2)
**Other	38 (40.0)
Missing	1 (1.1)

*‘Other’ included master of business administration (MBA) and masters in human resource management.

**The other category included individuals who were pursuing a master’s degree in business administration and master’s degree in management.

(N =95).

Seventy eight percent (74/95) of the respondents had no exposure; the remaining 22% (21/95) only had some exposure to cost effectiveness analysis. Nobody had a lot of exposure. Among those with a bachelor of medicine degree, only 19% (11/59) had some level of exposure to cost effectiveness analysis compared to 81% (48/59) who had no exposure to CEA. None (0/8) of those with master of medicine degree (MMED) had exposure to CEA and only 25% (3/12) of those with MS or MPH had some exposure to CEA (Table 2).

**Table 2:** **Respondents**’ **exposure to cost effectiveness analysis by their current level of training and work**-**place** (**N** = **94**)

**Training completed**	**Level of exposure**	
	**No exposure n (%)**	**Some exposure n (%)**
Bachelor of Medicine (MBChB) or MD	48 (81.4)	11 (18.7)
Post graduate (MMED)	8 (100.0)	0 (0.0)
MS/MPH	9 (75.0)	3 (25.0)
PhD	2 (100.0)	0 (0.0)
*Other	7 (53.9)	6 (46.2)
**Work-** **place**		
Ministry of Health	12 (80.0)	3 (20.0)
Tertiary/referral hospital	21 (65.6)	11 (34.4)
University	21 (77.8)	6 (22.2)
Non Governmental Organization	20 (100.0)	0 (0.0)
**Current training**		
Postgraduate training/MMED	14 (82.4)	3 (17.6)
MS/MPH	12 (75.0)	4 (25.0)
PhD in a health related field (e.g. epidemiology or Health Services Research)	17 (73.9)	6 (26.1)
*Other	31 (81.6)	7 (18.4)
Missing	0 (0.0)	1 (100.0)

*‘Other’ included master of business administration (MBA) and masters in human resource management.

Looking at exposure to CEA by work place also showed interesting findings. Among those working for the ministry of health, 12/15 (80%) had no CEA exposure. For tertiary/referral hospitals workers and university faculty, only 11/32 (34%) and 6/27 (22%) respectively, had some exposure to CEA. None (0/20) of those working for a non-governmental organization had any exposure to CEA.

Barriers to CEA identified by more than 50% of the participants were: lack of local experts in the field of CEA; lack of or limited local data; limited CEA training in schools; equity or ethical issues; lack of training grants incorporating CEA and lack of IT infrastructure. This meant the actual lack of or much limited supply of hardware such as computers and lack of or limited licenses to software packages such as TreeAge, which are used in economic evaluations such as CEA.

There was no statistical difference as to whether these were more of barriers in relation to policy or clinical practice. Participants also overwhelmingly agreed that improving these barriers will go a long way in increasing the use of CEA in policy and clinical practice (Table 3).

**Table 3:** **Barriers to CEA identified by the study**

**Barrier**	**Clinical practice n (%)**	**Policy formulation n (%)**	**Solving enhances use of CEA in clinical practice n (%)**	**Solving enhances use of CEA in policy formulation n (%)**
Lack of IT infrastructure e.g. hardware and software	72** (76.6)	65(69.9)	75 (80.7)	79 (85)
	N.S		N.S	
Lack of experts in the field of cost effectiveness analysis	81 (87.1)	76 (81.7)	89 (95.7)	89 (95.7)
	N.S		N.S	
Lack of or limited local data	85 (91.4)	82 (88.2)	88 (94.6)	90 (96.8)
	N.S		N.S	
Limited CEA training in schools	89 (95.7)	86 (92.5)	89 (95.7)	89 (95.7)
	N.S		N.S	
Fairness and ethical concerns	47 (50.5)	49 (54.1)	N/A	N/A
	N.S		N/A	
Lack of training grants incorporating CEA	61 (65.6)	63 (67.7)	73 (78.5)	74 (79.6)
	N.S		N.S	

N.S*:* Chi*-*square goodness of fit test showed that the observed proportions of agreeing to a given barrier were not statistically different whether the barrier concerned policy or clinical practice*.*

N*/*A*:* Not applicable*.*

**Is based on N *=*94*,* all the other proportions in the table are based on N *=*93. These are less than 95 because of missing data resulting from participants not responding to certain questions*.*

Majority of the respondents overwhelmingly agreed that CEA should play an important role in policy and clinical practice. Again there was no statistical difference as to whether perception of the importance of CEA was in relation to policy or clinical practice (Table 4).

**Table 4:** **Respondents**’ **perceived importance of cost effectiveness analyses in policy and clinical practice**

**Respondent’** **s perception of importance of CEA**	**In clinical practice n (%)**	**In policy formulation n (%)**
CEA should play an important role	92 (96.8)	91 (95.8)
	N.S	
Your colleagues think that CEA should play an important role	69 (72.6)	74** (78.7)
	N.S	

N.S*:* Chi*-*square goodness of fit test showed that the observed proportions of agreeing to the importance of CEA were not statistically different whether the importance was in relation to policy or clinical practice*.*

**Is based on N *= *94 because of a missing data point*,* all the other proportions in the table are based on N *= *95*.*

Among those who had no exposure to CEA, 43% (32/74) had a lot of interest in conducting CEA compared to 57% (42/74) who did not have a lot of interest in conducting CEA. Among those who had some exposure to CEA, 30% (6/20) had a lot of interest in conducting CEA compared to 70% (14/20) who did not have a lot of interest in conducting CEA.

The picture was slightly different when we looked at CEA exposure versus interest in learning to conduct CEA. Although not statistically significant, respondents who had no exposure to CEA compared to those who had some exposure were slightly less likely to be interested in learning to conduct CEA, 58% (43/74) vs. 65% (13/20), p = 0.08.

In general, most of the respondents frequency of using CEA in making clinical or policy decisions ranged between sometimes to rarely or never (Table 5).

**Table 5:** **Respondents**’ **frequency of use of cost effectiveness analysis**

	**Always n (%)**	**Sometimes n (%)**	**Rarely or never n (%)**
How often have you been involved in doing CEA?**	3 (3.2)	24 (25.3)	68 (71.6)
How often have you (personally) had to interpret CEA during your practice or decision making?	6 (6.4)	41 (43.6)	47 (50.0)
How frequently do you read an article (s) that compares the cost effectiveness of two or more interventions?	2 (2.1)	50 (53.2)	42 (44.7)
In the past 12 months, have you considered cost effectiveness analysis results when making decisions during your clinical practice?	10 (10.6)	39 (41.5)	45 (47.9)
In the past 12 months, have you considered cost effectiveness analysis results when formulating any policies?	10 (10.7)	32 (34)	52 (53.3)
How often do you involve CEA in discussing policy or practice at your school or place of work?	8 (8.5)	50 (53.2)	36 (38.3)
How often have you been involved in reviewing CEA?	2 (2.1)	26 (27.7)	66 (70.2)

**Proportions based on N *=*95*,* due to one missing data value all the other proportions in the table are based on N *= *94*.*

## Discussion

In the resource-constrained countries of Sub-Saharan Africa (SSA), where policy and decision makers are frequently faced with tough decisions regarding resource allocation, cost effectiveness analysis (CEA) can be a useful tool for optimal allocation of scarce resources. This study shows that the health care actors in Uganda perceive cost effectiveness analysis as an important part of the decision making process.

This study identified the lack of exposure to CEA and the lack of trained individuals to conduct CEA as the two main barriers to CEA playing a substantial role in health care decision making in Uganda. Just like in Thailand and other developing countries [8], the majority of the health policy decision makers in Uganda are medically trained. Systemic introduction of CEA methods into medical training is necessary, and also requires the creation of training grants for CEA.

Lessons learned from the approaches that developed nations have used to increase the knowledge and skills to carry out CEA should be applied to developing countries. Noteworthy is the fact that the approaches adopted should be modified in ways which make them appropriate in the local context. This will enhance the impact of CEA in the decision making process. A review by Alan Stewart, Jordana K. Schmier, & Bryan R. Luce, 1999 [9] has documented some of these strategies.

Jauregui et al., 2011, describes the Pan-American Health Organization (PAHO) ProVac Initiative’s approach. This initiative was in response to the request by country managers of vaccine programs for additional technical support in the use of cost-effectiveness analysis and other forms of economic evaluations of competing interventions. Its implementation inter-alia resulted into new models for cost-effectiveness analysis; innovative data collection tools; CEA trainings at regional levels; technical support to individual nations that ask for it and development of web-based based technical support systems [10]. These lessons can be applied to SSA.

In addition, the WHO-CHOICE(CHOosing Interventions that are Cost-Effective) project provides key information needed to standardize CEA methods, and also takes a sectoral perspective where the costs and effectiveness of a wide range of interventions are compared in order to identify those that maximize health from a given set of resources. The WHO-CHOICE project also assembles regional databases on the costs, and impact on population health. It also provides cost-effectiveness of key health interventions, as well as a contextualization tool, enabling adaption of regional results to the country level [3].

In this study, less than one in four had any exposure to CEA, including the subset of Ministry of Health employees. Furthermore, more than 80% identified lack of local experts as a barrier to the use of CEA. Similar findings have been observed among policy makers in Thailand, where only 29% were able to define the concept of economic evaluations [8]. Following these findings, interventions such as capacity building using various approaches like short-term trainings, workshops, and conferences about CEA methodologies and approaches have been widely implemented in Thailand [5]. With necessary modifications to make them locally appropriate, these should be adopted in Uganda and other developing or poorest countries.

This study identified other barriers similar to those identified by a range of policymakers in a Thailand study [5]. Among these is the lack of infrastructure and physical tools like computers and associated software.

In addition, there is limited local data and policy makers are routinely using clinical data from other countries.

In Thailand, the Health Intervention and Technology Assessment Program has adopted certain principles, such as technical excellence and policy relevance to enhance the utilization of its research findings by policy makers and other stakeholders [5].

There is a need for establishing cost effectiveness analysis institutes in SSA to guide research involving CEA and also carry out capacity building activities. While some developing nations like Thailand have been able to do this on their own, for the extremely impoverished countries of Sub-Saharan Africa, it may be necessary to establish a regional body. This may start by establishing a think tank comprised of key actors in health care decision making and experts in local institutions such as the economic policy research center in Uganda, the Kenya Medical Research Institute, and the Botswana institute of social policy to guide capacity building for CEA in SSA. This approach should be part of an overall strategy and can help define a better, sustainable process for increasing awareness and capacity to conduct the analysis.

Furthermore, fairness and ethical concerns were also cited as significant barriers to CEA. Societal values such as equity may sometimes conflict with the conclusions of CEA evaluations. For example, providing universal access to second-line antiretroviral therapy (ART) for treating HIV/AIDS patients may not prove to be as cost-effective, but may be preferable to society [11].

On the other hand, the fact stands that affordability of an otherwise cost effective intervention can be a barrier to wide spread use of knowledge gained from CEA. The additional cost of the program (affordability) when fully implemented may be prohibitive for a given country [12,13] despite a favorable ICER per outcome for one person.

A high proportion of those who had no CEA exposure had a lot of interest in learning to conduct it, yet only 35% of those who had some exposure had a lot of interest in learning to conduct CEA. This may be because people who have had some exposure to CEA may better realize how complex CEA can be, and may prefer not to make the investment of time to learn to do it themselves. They still found it important to do CEA. Indeed, developing capacity to conduct CEAs will likely require the teaching of these methods in quantitative health-related disciplines such as epidemiology and biostatistics and might be augmented by the Ministry of Health requiring staff be knowledgeable and skilled in CEA.

Also noteworthy was the finding that none of the twenty individuals employed by NGOs reported any exposure to CEA. This may be because many NGOs have specific well-funded missions, and are not conducting CEA and formulating policy.

It is worth noting that although CEA may be essential for guiding resource allocation, its use might be limited in countries like Uganda, where resource allocation for health care is governed by a number of stakeholders including government and development partners such as Global fund, that may be interested in funding specific programs, e.g., Malaria, HIV/AIDS, medicines and diagnostics etc. [14].

In such settings, the lack of a single unitary agency e.g., the ministry of health that governs resource allocation limits the use of CEA tools to inform resource allocation at a sector level. This creates inefficiencies. However, within a sector program e.g., HIV/AIDS Anti-retro-viral drugs, there might be circumstances where CEA tools might be applicable.

The biggest limitation of this study is the small sample size, with limited power to detect significant differences between subgroups. However, the sample size was adequate to support the exploratory aims of our study which will guide future studies and interventions. Having mentioned that however, participants in this study belonged to categories that were identified as the main health care actors in Uganda [6] and elsewhere [15], therefore the information they provide is assumed to be both relevant and reliable. As with any self-administered questionnaire, we also cannot rule out the possibility of poor question interpretation [16]. Nevertheless, systematic analysis of our questionnaire results did not identify any specific questions that appeared to be interpreted illogically.

## Conclusions

This study demonstrates that despite substantial interest, significant barriers have to be dealt with if cost effectiveness analysis is to have a significant impact on policy and practice in Sub-Saharan Africa. More locally generated data is needed, as is improved infrastructure, and increased local expertise. The study also highlights the unfortunate reality that despite acknowledgement of the importance of CEA and interest in learning to conduct it, very few health care actors in Uganda have sufficient exposure to CEA for these methods to impact policy. If cost effectiveness analysis is to take root in Sub-Saharan African countries like Uganda, there must be concerted effort to increase exposure to CEA at all levels of training.

Since political pressure also plays a role in decision making [8], there is a need for effective dialogue with all the involved stakeholders, particularly political representatives so that they understand the appropriate role for CEA in policy making.

## References

[1] Singer ME (2008). Cost-Effectiveness Analysis: Developing Nations Left Behind. Pharmacoeconomics.

[2] The World Bank: **Africa’s Population Set to Double by 2036.** 2010. [cited 2014 June 15]; Available from: http://go.worldbank.org/X6PO5E1410.

[3] Hutubessy R, Chisholm D, Edejer T (2003). WHO-CHOICE. Generalized cost-effectiveness analysis for national-level priority-setting in the health sector. Cost Effectiveness and Resour Allocation.

[4] Goodman CA, Mills AJ (1999). The evidence base on the cost-effectiveness of malaria control measures in Africa. Health Policy Plan.

[5] Tantivess S, Teerawattananon Y, Mills A (2009). Strengthening Cost-Effectiveness Analysis in Thailand through the Establishment of the Health Intervention and Technology Assessment Program. Pharmacoeconomics.

[6] Kapiriri L, Arnesen T, Norheim OF (2004). Is cost-effectiveness analysis preferred to severity of disease as the main guiding principle in priority setting in resource poor settings? The case of Uganda. Cost Eff Resour Alloc.

[7] Babar Z-U-D, Scahill S (2010). Is there a role for pharmacoeconomics in developing countries?. Pharmacoeconomics.

[8] Teerawattananon Y, Russell S (2008). A Difficult Balancing Act: Policy Actors’ Perspectives on Using Economic Evaluation to Inform Health-Care Coverage Decisions under the Universal Health Insurance Coverage Scheme in Thailand. Value Health.

[9] Stewart A, Schmier JK, Luce BR (1999). Economics and cost-effectiveness in evaluating the value of cardiovascular therapies. A survey of standards and guidelines for cost-effectiveness analysis in health care. Am Heart J.

[10] Jauregui B, Sinha A, Clark AD, Bolanos BM, Resch S, Toscano CM, Matus CR, Andrus JK (2011). Strengthening the technical capacity at country-level to make informed policy decisions on new vaccine introduction: Lessons learned by PAHO’s ProVac Initiative. Vaccine.

[11] Cleary SM, McIntyre D, Boulle AM (2008). Assessing efficiency and costs of scaling up HIV treatment. AIDS.

[12] Weintraub WS, Cohen DJ (2009). The Limits of Cost-Effectiveness Analysis. Circ Cardiovasc Qual Outcomes.

[13] Babigumira JB, Levin A, Burgess C, Garrison LP, Bauch CT, Braka F, Mbabazi WB, Nabyonga JO, Simons E, Dabbagh A (2011). Assessing the Cost-Effectiveness of Measles Elimination in Uganda: Local Impact of a Global Eradication Program. J Infect Dis.

[14] Niessen LW, Bridges J, Lau BD, Wilson RF, Sharma R, Walker DG, Frick KD, Bass EB (2012). Assessing the Impact of Economic Evidence on Policymakers in Health Care—A Systematic Review. In *ᅟ*.

[15] Grinten V (2000). Actors in priority setting: Intended roles and actual behaviour. In *ᅟ*.

[16] Bowling A (2005). Mode of questionnaire administration can have serious effects on data quality. J Public Health (Oxf).

